# Talin Modulation by a Synthetic *N*-Acylurea Derivative Reduces Angiogenesis in Human Endothelial Cells

**DOI:** 10.3390/ijms18010221

**Published:** 2017-01-22

**Authors:** I-Rang Lim, Hyung Joon Joo, Minseon Jeong, Jong-Ho Kim, Seung-Cheol Choi, Chungho Kim, Jong-Wha Jung, Soon Jun Hong

**Affiliations:** 1 Department of Cardiology, Cardiovascular Center, College of Medicine, Korea University, Seoul 02841, Korea; irang.lim@gmail.com (I.-R.L.); drjoohj@gmail.com (H.J.J.); mecey@naver.com (J.-H.K.); choisc86@gmail.com (S.-C.C.); 2 College of Pharmacy, Research Institute of Pharmaceutical Sciences, Kyungpook National University, Daegu 41566, Korea; dha66@naver.com (M.J.); jungj@knu.ac.kr (J.-W.J.); 3 Department of Life Sciences, Korea University, Seoul 02841, Korea; chungho@korea.ac.kr

**Keywords:** KCH-1521, talin, focal adhesion, talin modulator, anti-angiogenesis

## Abstract

Talin is a focal adhesion protein that activates integrins and recruits other focal adhesion proteins. Talin regulates the interactions between integrins and the extracellular matrix, which are critical for endothelial cells during angiogenesis. In this study, we successfully synthesized a novel talin modulator, *N*-((2-(1*H*-indol-3-yl)ethyl)carbamoyl)-2-(benzo[*d*][1,3]dioxol-5-yloxy)acetamide, referred to as KCH-1521. KCH-1521 was determined to bind talin and modulate downstream signaling molecules of talin. After 24 h of treatment, KCH-1521 changed the cell morphology of human umbilical vein endothelial cells (HUVECs) and reduced focal adhesion protein expression including vinculin and paxillin. Talin downstream signaling is regulated via focal adhesion kinase (FAK), kinase B (AKT), and extracellular signal-regulated kinase (ERK) pathways, however, treatment with KCH-1521 decreased phosphorylation of FAK, AKT, and ERK, leading to reduction of cell proliferation, survival, and angiogenesis. Interestingly, the expression of various angiogenic genes was significantly decreased after treatment with KCH-1521. Also, in vitro tube forming assay revealed that KCH-1521 reduced angiogenic networks in a time-dependent manner. To investigate the reversibility of its effects, KCH-1521 was removed after treatment. HUVECs recovered their morphology through rearrangement of the cytoskeleton and the expression of angiogenic genes was also recovered. By further optimization and in vivo studies of KCH-1521, a novel drug of talin modulation could be used to achieve therapeutic anti-angiogenesis for vascular diseases and cancers.

## 1. Introduction

Integrin inside-out activation is mainly regulated by the intracellular protein talin. Integrin is fully activated when outside-in signaling is activated in response to the extracellular matrix (ECM) such as fibronectin, laminin, and collagen [1]. These stimulate numerous biological processes such as cell adhesion, survival, proliferation, and metastasis [2]. Overexpression of talin1 increased cell adhesion, migration, and invasion of human prostate cancer cells. Loss of talin1 resulted in a significant inhibition of prostate cancer cell migration and invasion in vitro, and reduced metastatic tumor lesions in vivo [3]. In contrast, mice lacking the entire talin2 coding sequence were viable and fertile only with a mild dystrophic phenotype [4].

Talin is a critical focal adhesion (FA) regulator that links integrin and the actin cytoskeleton and induces FA formation. Talin contains the binding sites for integrin β3 [5], actin, vinculin, and focal adhesion kinase (FAK), as well as specifically multiple vinculin-binding sites that are pivotal for FA localization of vinculin [6]. In response to integrin-talin interaction, FAK is activated via autophosphorylation on the tyrosine 397 residues [7]. Consequently, it leads to activation of its kinase function and downstream signaling pathways such as phosphoinositide 3-kinase (PI3K)/protein kinase B (AKT) and mitogen-activating protein kinase (MAPK)/extracellular signal-regulated kinase (ERK) pathways and paxillin resulting in cytoskeletal and morphological changes [7,8]. Overexpression of talin1 in prostate cancer cells resulted in activation of AKT and MAPK under collagen and fibronectin attachment conditions [3].

Several recent studies have indicated that talin1 may have potential as a novel prognostic marker and therapeutic target. High expression of talin1 was significantly associated with poor distant metastasis-free survival and low five-year overall survival in patients with nasopharyngeal carcinoma, especially in advanced stages [9]. The clinical research of hepatocellular carcinoma revealed that talin1 was significantly upregulated according to tumor progression [10]. In addition, talin1-lacking platelets failed to activate integrin and platelet aggregation, resulting in a severe hemostatic defect and resistance to thrombosis [11].

Angiogenesis is a complex process regulated by numerous receptors, growth factors, ECM-cell and cell-cell interactions. Endothelial cells need to migrate, invade the ECM, and form capillary structures during angiogenesis. Therefore, the integrin-mediated interaction with ECM is critical in the regulation of angiogenesis [12]. In particular, αVβ3 integrin has been reported as an important regulator of angiogenesis [13,14] and endothelial functions such as spreading and migration in human umbilical vein endothelial cells (HUVECs) [15]. Reduction of talin inhibited the conformational rearrangement of integrin, suggesting that talin is required for activation of αVβ3 integrin [16]. Disruption of talin in endothelial cells impaired organization into vessels, leading to embryonic death at gastrulation [17]. Moreover, knockdown of talin using small interference RNA (siRNA) altered HUVECs into arborized or elongated shapes that showed a significantly shorter trajectory and reduced velocity, suggesting that talin is required to maintain the normal morphology and migration [18].

In the present study, we synthesized a novel talin modulator, *N*-((2-(1*H*-indol-3-yl)ethyl)carbamoyl)-2-(benzo[*d*][1,3]dioxol-5-yloxy)acetamide, referred to as KCH-1521, that is an *N*-acylurea derivative, and explored its effects and related mechanisms on HUVECs.

## 2. Results

### 2.1. Synthesis and Chemical Properties of KCH-1521

**Figure 1:**
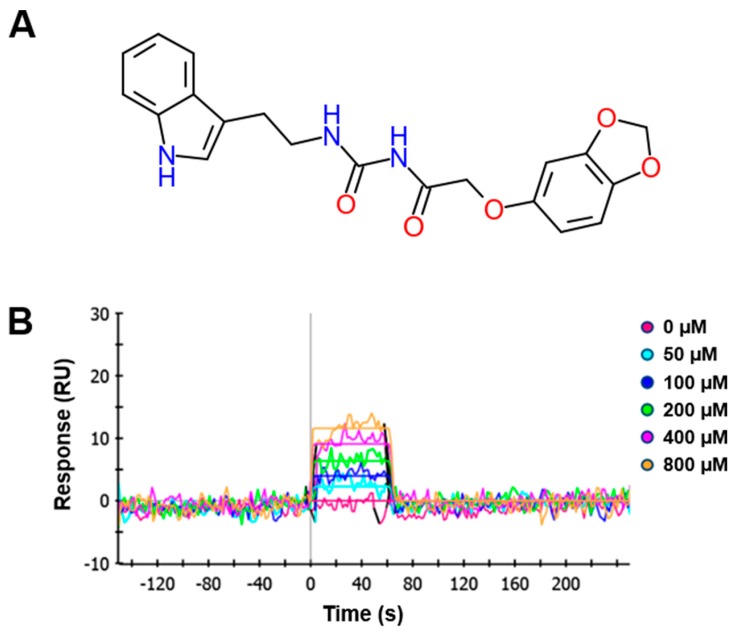
Properties of a novel talin modulator, KCH-1521. (**A**) Chemical structure of *N*-((2-(1*H*-indol-3-yl)ethyl)carbamoyl)-2-(benzo[*d*][1,3]dioxol-5-yloxy)acetamide (KCH-1521); (**B**) Representative sensorgram from surface plasmon resonance (SPR) analysis of talin-KCH-1521 binding. The binding of KCH-1521 with talin fixed on a sensor chip was observed in a dose-dependent manner with a K_D_ value of 3.01 × 10^−4^ M. RU, resonance units.

KCH-1521, *N*-((2-(1*H*-indol-3-yl)ethyl)carbamoyl)-2-(benzo[*d*][1,3]dioxol-5-yloxy)acetamide is one of many synthetic *N*-acylurea derivatives which are designed to modulate talin (Figure 1A). KCH-1521 was selected for in vitro cellular evaluation after screening (unpublished data). Binding of synthesized KCH-1521 to talin was confirmed by surface plasmon resonance (SPR) analysis using purified glutathione-*S*-transferase-fused talin head domain at various concentrations of KCH-1521. The sensorgram of SPR showed that each of the curves represented six different concentrations of KCH-1521 (0, 50, 100, 200, 400, and 800 μM). As the concentration of KCH-1521 increased, the response units (RU) increased and were detected even at relatively low concentrations of KCH-1521. In addition, KCH-1521 successfully bound to talin with stability and reversibly bound to talin at all concentrations (Figure 1B).

### 2.2. Effects of KCH-1521 on Cell Viability

To evaluate the expression of talin1 and talin2 in HUVECs, semi-quantitative PCR was performed and normal human dermal fibroblasts (NHDFs) were used as a positive control for talin abundance [3]. Both talin1 and talin2 were detected in HUVECs (Supplementary Figure S1A). In accordance with this, the immunofluorescence staining manifested that talin was expressed through the whole cell and especially localized to FA in the peripheral region of HUVECs (Supplementary Figure S1B, left), and the expression of talin was maintained even in higher passages (Supplementary Figure S1B, right).

**Figure 2:**
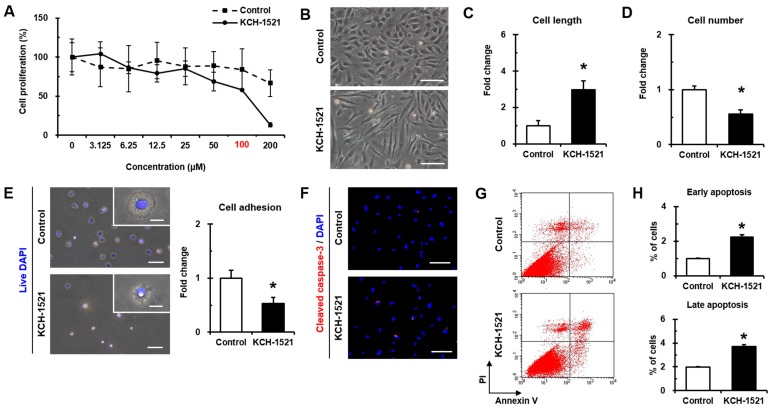
Characterization of the effects of KCH-1521 on human umbilical vein endothelial cells (HUVECs). (**A**) WST-1 assay indicating dose-dependent cell proliferation of HUVECs. The untreated condition (0 μM) was regarded as a 100% cell proliferation rate. The experimental concentration of KCH-1521 was 100 μM (red); (**B**) representative phase-contrast images of HUVECs after 24 h of treatment with KCH-1521 at a concentration of 100 μM. Scale bars = 100 μm; (**C**) cell length of single cells measured in terms of long axis; (**D**) Cell number quantified after 24 h of treatment; (**E**) representative overlapped immunofluorescence and phase-contrast images after 10 min of attachment, and quantification of attached cells. Nuclei were stained with live DAPI (4′,6-diamidino-2-phenylindole; blue). Scale bars = 100 μm for the large panels, and 20 μm for the magnified insets; (**F**) Representative immunofluorescence images showing cleaved caspase-3 (red) expression in the control and KCH-1521-treated cells. Nuclei were stained with DAPI (blue). Scale bars = 100 μm; (**G**) flow cytometric analyses of Annexin V and propidium iodide (PI) staining in the control and KCH-1521-treated cells; (**H**) quantification of early (Annexin V-positive and PI-negative) and late (Annexin V-positive and PI-positive) apoptotic cell ratio. Data shown represent the mean ± SD of three independent experiments (* *p* < 0.05).

Effects of different concentrations of KCH-1521 on the proliferation of HUVECs were evaluated by WST-1 assay to determine the appropriate concentration. As shown in Figure 2A, KCH-1521 showed a dose-dependent decrease in proliferation, and the half maximal inhibitory concentration of KCH-1521 was 100 μM (58.17%), compared to the untreated condition (0 μM) that was regarded as 100%. Therefore, this concentration of KCH-1521 was used in all experiments in this study. After 24 h of treatment with KCH-1521, HUVECs showed morphological changes with a tendency to sharpen and form into elongated shapes, whereas in the control, the endothelial cell morphology was maintained (Figure 2B). The cell length in the long axis was increased by KCH-1521 (2.98-fold) compared to the control (Figure 2C). Consistent with Figure 2A, the number of cells was significantly decreased by treatment with KCH-1521 at the half maximal inhibitory concentration (0.55-fold) compared to the control, indicating that KCH-1521 decreased cell proliferation in HUVECs (Figure 2D). In addition, treatment of HUVECs with KCH-1521 significantly decreased the number of live and adherent cells compared to the control, indicating the reduction of cell adhesion by KCH-1521 (Figure 2E). Interestingly, there were differences in spreading morphology between the control and KCH-1521-treated cells (white boxes in Figure 2E). Furthermore, KCH-1521 showed higher expression of cleaved caspase-3, indicating activation of caspase-3 and increased apoptosis compared to the control (Figure 2F). Flow cytometric analysis of Annexin V/propidium iodide (PI) staining revealed significantly increased early apoptotic and late apoptotic cells caused by KCH-1521 (2.23-fold and 1.87-fold, respectively) compared to the control (Figure 2G,H).

### 2.3. Decreased Angiogenic Potential of HUVECs by KCH-1521

**Figure 3:**
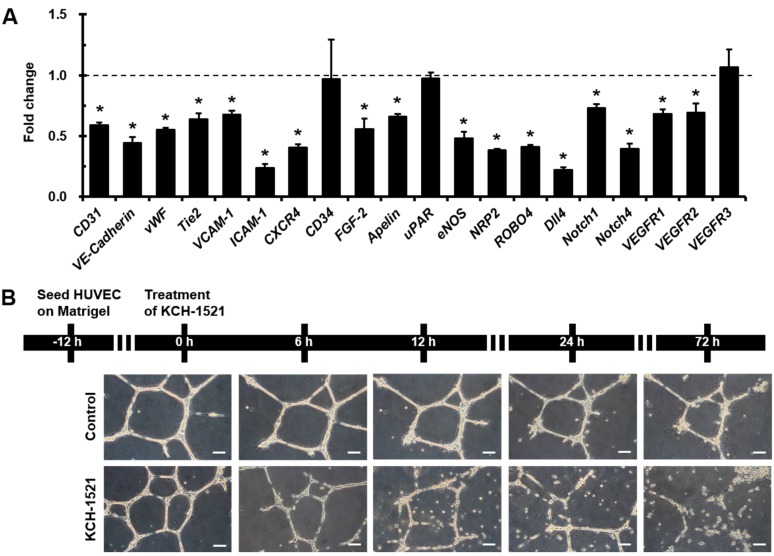
In vitro anti-angiogenic effects of KCH-1521 on HUVECs. (**A**) Relative changes in angiogenic gene (*CD31*, *VE-Cadherin*, *vWF*, *Tie2*, *VCAM-1*, *ICAM-1*, *CXCR4*, *CD34*, *FGF-2*, *Apelin*, *uPAR*, *eNOS*, *NRP2*, *ROBO4*, *Dll4*, *Notch1*, *Notch4*, *VEGFR1*, *VEGFR2*, and *VEGFR3*) expression in KCH-1521-treated cells compared to the control, as determined by real-time PCR. The dotted line represents the relative value of the control; (**B**) schematic diagram and representative phase-contrast images of in vitro tube forming assay of the control and KCH-1521-treated cells on Matrigel. Scale bars = 100 μm. Data shown represent the mean ± SD of three independent experiments (* *p* < 0.05).

Given that talin modulation affected the cellular characteristics of HUVECs, we investigated the effects of KCH-1521 on angiogenesis in HUVECs. Interestingly, treatment with KCH-1521 significantly reduced the expression of angiogenic genes such as *CD31* (0.59-fold), vascular endothelial-Cadherin (*VE-Cadherin*; 0.44-fold), von Willebrand factor (*vWF*; 0.55-fold), *Tie2* (0.64-fold), vascular cell adhesion molecule-1 (*VCAM-1*; 0.68-fold), intercellular adhesion molecule-1 (*ICAM-1*; 0.24-fold), C-X-C chemokine receptor type 4 (*CXCR4*; 0.41-fold), fibroblast growth factor-2 (*FGF-2*; 0.56-fold), *Apelin* (0.66-fold), endothelial nitric oxide synthase (*eNOS*; 0.48-fold), and neuropilin 2 (*NRP2*; 0.39-fold) compared to the control. Additionally, the expression of roundabout guidance receptor 4 (*ROBO4*; 0.41-fold), delta like ligand (*Dll4*; 0.22-fold), *Notch1* (0.73-fold), and *Notch4* (0.39-fold) was significantly decreased in KCH-1521-treated cells compared to the control. KCH-1521-treated cells presented significantly lower expression of vascular endothelial growth receptor 1 (*VEGFR1*; 0.68-fold) and *VEGFR2* (0.70-fold) than the control. However, the expression of *CD34* (0.97-fold), urokinase receptor (*uPAR*; 0.98-fold), and *VEGFR3* (1.07-fold) in KCH-1521 was similar to that in the control (Figure 3A). We also examined the in vitro anti-angiogenic effects of KCH-1521. As expected, 12 h after plating HUVECs on Matrigel, a time-dependent reduction in tube and network formation was seen in KCH-1521-treated cells compared to the control (Figure 3B).

### 2.4. Reversible Effects of KCH-1521 on HUVEC Morphology

**Figure 4:**
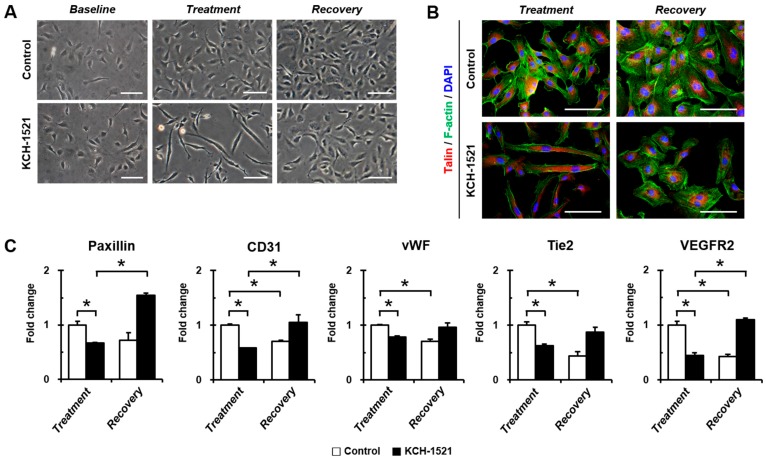
Reversibility of KCH-1521 on HUVECs. (**A**) Representative phase-contrast images indicating cell morphology (*Baseline*), morphological change (*Treatment*) after 24 h of treatment, and reversibility (*Recovery*) after 24 h of KCH-1521 removal compared to the control. Scale bars = 100 μm; (**B**) representative immunofluorescence images of talin (red) and F-actin (green) in the control and KCH-1521. Nuclei were stained with DAPI (blue). Scale bars = 100 μm; (**C**) real-time PCR analyses showing recovery of *paxillin*, *CD31*, *vWF*, *Tie2*, and *VEGFR2* gene expression at *Recovery* compared to *Treatment* in KCH-1521. Data shown represent the mean ± SD of three independent experiments (* *p* < 0.05). Scale bars in (**A**,**B**) = 100 μm.

To determine whether KCH-1521 effects are reversible, HUVECs were incubated in the culture medium containing either dimethyl sulfoxide (DMSO) or KCH-1521 for 24 h (*Treatment*) after 24 h of attachment (*Baseline*). DMSO or KCH-1521 was removed and HUVECs were cultured in fresh culture medium for an additional 24 h (*Recovery*). The morphology of HUVECs changed into elongated and fibroblast-like shapes after treatment with KCH-1521 compared to *Baseline*. Upon removal of KCH-1521, HUVECs restored their endothelial morphology, as shown in the phase-contrast image of *Recovery* (Figure 4A). Interestingly, immunofluorescence staining of F-actin revealed that the actin cytoskeleton was disrupted by KCH-1521 at *Treatment* compared to the well-organized cytoskeleton seen in the control. These results suggest that KCH-1521 also affected the interaction between talin and actin. At *Recovery*, HUVECs were almost completely reverted to a cytoskeletal arrangement with the original morphology that was similar to that of the control (Figure 4B). Indeed, analysis of *paxillin* expression at the mRNA level showed that the reduced expression by KCH-1521 treatment was increased at *Recovery* (1.54-fold) compared to the control at *Treatment*. Removal of KCH-1521 for 24 h led to restoration of angiogenic gene expression such as *CD31*, *vWF*, *Tie2*, and *VEGFR2* (Figure 4C). Consequently, talin modulation induced changes in cell morphology, cytoskeleton arrangement, and angiogenic gene expression that were restored by removal of KCH-1521. As shown in Figure 4 which corresponds to the SPR analysis (Figure 1B), KCH-1521 is reversible in its binding to and modulation of talin.

### 2.5. Reduction of the Talin-Mediated Signaling Pathway by KCH-1521

**Figure 5:**
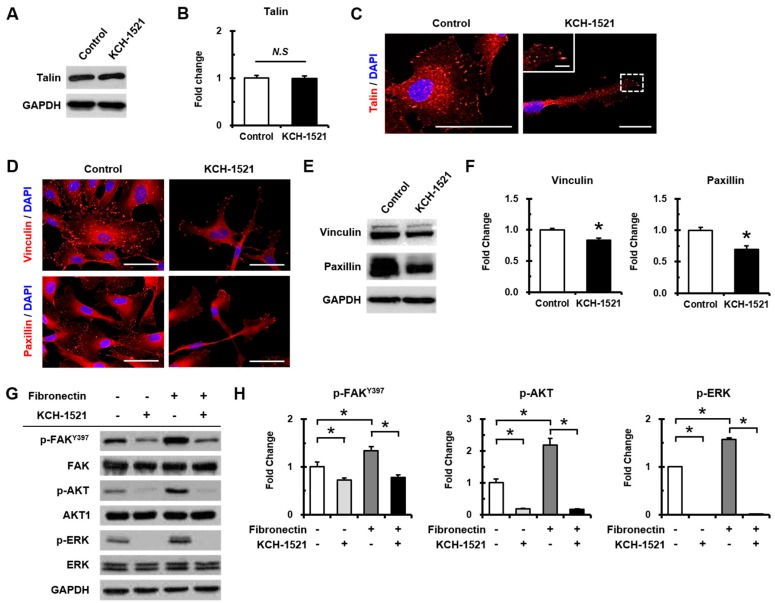
Effects of KCH-1521 on focal adhesion molecules. (**A**) Western blot of talin expression after 24 h of treatment with dimethyl sulfoxide (DMSO) as the control or KCH-1521; (**B**) quantification of talin expression normalized to glyceraldehyde 3-phosphate dehydrogenase (GAPDH); (**C**) representative immunofluorescence images of talin (red) expression in the control and KCH-1521-treated cells. The magnified inset showing talin expression in focal adhesion (FA). Nuclei were stained with DAPI (blue). Scale bars = 50 µm for the large panels and 20 µm for the magnified inset; (**D**) representative immunofluorescence images of vinculin (top panel, red) and paxillin (bottom panel, red) expression in the control and KCH-1521-treated cells. Nuclei were stained with DAPI (blue). Scale bars = 50 µm; (**E**) Western blot of vinculin and paxillin expression in the control and KCH-1521-treated cells; (**F**) quantification of vinculin and paxillin expression normalized to GAPDH; (**G**) Western blot of p-FAK^Y397^, FAK, p-AKT, AKT, p-ERK, ERK, and GAPDH expression after 6 h of adherence to fibronectin; (**H**) quantification of p-FAK, p-AKT, and p-ERK expression normalized to total FAK, AKT, and ERK, respectively. Data shown represent mean ± SD of three independent experiments (* *p* < 0.05; *N.S*, not significant).

Binding of KCH-1521 to talin did not affect the expression of talin protein (Figure 5A, B). KCH-1521 led to less spanned expression of talin through the whole cell compared to the control, and to localized expression in peripheral regions (Figure 5C). Upon binding to integrin, talin recruited FA-related proteins including vinculin and paxillin, therefore, analyses of FA proteins were performed in talin-modulated HUVECs by KCH-1521. Immunofluorescence staining showed that KCH-1521 decreased both vinculin and paxillin expression that were specifically localized in the FA via talin modulation (Figure 5D). Indeed, we found that KCH-1521 significantly reduced the expression of vinculin (0.83-fold) and paxillin (0.69-fold) at the protein level compared to the control (Figure 5E,F). To investigate the talin-mediated integrin signaling pathways that could be regulated by KCH-1521, the activities of multiple signaling molecules including FAK, AKT, and ERK were examined in the presence of fibronectin as ECM (Figure 5G). Integrin activation under fibronectin-attached conditions subsequently upregulated talin and significantly increased the phosphorylation of FAK on tyrosine 397 (FAK^Y397^), AKT, and ERK (1.34-fold, 2.18-fold, and 1.57-fold, respectively) compared to fibronectin-uncoated conditions. Modulation of talin by KCH-1521 significantly reduced the phosphorylation of FAK, AKT, and ERK (0.73-fold, 0.18-fold, and 0.00-fold respectively), even under conditions of fibronectin-induced integrin activation (0.80-fold, 0.16-fold, and 0.01-fold, respectively) (Figure 5H).

## 3. Discussion

The novelties of the present study were that (1) we successfully synthesized a new talin modulator, KCH-1521; (2) we described the first small molecule, KCH-1521 that binds to talin and down-regulated subsequent FA-related molecules; and (3) we showed the in vitro anti-angiogenic effects of KCH-1521 in HUVECs.

Synthesized KCH-1521 at the concentration of 100 μM was dissolved in 1% (*v*/*v*) DMSO that was used as the vehicle control in our study. We found that there was no significance in absorbance units between the untreated condition (0 μM) and 1% (*v*/*v*) DMSO (indicated as 100 μM in Figure 2A). This is supported by the recent study showing that treatment with 1% (*v*/*v*) DMSO for 24 h showed no significant increases in the number of pyknotic nuclei and apoptotic cells compared to the untreated control. Also, there was no significance in the decrease in cell viability between 0% and 1% (*v*/*v*) DMSO [19]. Moreover, Sumida et al. [20] revealed that no cytotoxic effects of DMSO were observed up to the 2% (*v*/*v*) by lactate dehydrogenase (LDH) activity tests in both human and rat hepatocytes after 24 h of treatment. Therefore, we considered that there was no significant cytotoxicity of 1% (*v*/*v*) DMSO on HUVECs when used as the vehicle control in this study.

Integrin signaling has been implicated in various cell physiological processes [2]. In platelets, integrin mediates platelet adhesion and aggregation, therefore, integrin modulation has become an attractive strategy for antiplatelet effects [21]. Also, endothelial cell integrin regulates numerous cell functions including angiogenic sprouting [22]. Recently, small molecules regulating integrin signaling such as FAK inhibitors have been developed and extensively studied in various cancer cells, demonstrating both in vitro and in vivo anti-tumor effects [23,24]. However, small molecules that modulate other FA molecules especially talin have not yet been reported.

In vertebrates, there are two isoforms of talin, and endothelial cells express talin1 and little or no talin2. HUVECs transfected with talin1 siRNA were elongated and showed a loss of actin stress fibers and actin accumulation in the lamellae compared to those transfected with control RNA [18]. In accordance with a previous report, HUVECs used in our study were positive for talin1 and talin2 (Supplementary Figure S1A) and talin modulation by KCH-1521 induced morphological changes in the form of elongation in shape and increases in the cell length of HUVECs (Figure 2B,C). Although there was no significant difference in the amount of talin protein between the control and KCH-1521 treatment (Figure 5A,B), KCH-1521 led to less spanned expression of talin (Figure 5C) and talin-binding F-actin (Figure 4B), which were considered to be involved in changes of cell morphology. These findings indicate that modulation of talin by KCH-1521 may have similar effects to those of talin knockdown using siRNA as reported by Kopp et al. [18].

Talin-mediated integrin activation triggers recruiting of multiple FA molecules and the inducing of intracellular signaling cascades [25]. FAK is a downstream molecule of integrin and is essential for focal adhesion-dependent signals [26]. FAK-AKT signaling, in which AKT is one of the key downstream molecules of the PI3K pathway, is involved in cell survival and apoptosis [8,25,27]. In this study, treatment with KCH-1521 resulted in significant decreases in phosphorylation of FAK and AKT (Figure 5G,H) and increases in apoptosis by cleaved caspase-3 (Figure 2F) and Annexin V/PI staining (Figure 2G,H) compared to the control. Furthermore, FAK-ERK signaling of the intracellular MAPK pathway was associated with cell proliferation [8,26]. In HUVECs, we also noted the reduced phosphorylation of ERK (Figure 5G,H) and dose-dependent decrease in proliferation by KCH-1521 (Figure 2A). Consequently, KCH-1521 led to reduced proliferation and increased apoptosis via suppression of the intercellular signaling pathways of integrin in HUVECs.

Many previous studies on angiogenesis using small molecules have been focused on receptor tyrosine kinases such as VEGFR, FGF receptor (FGFR), or EGF receptor (EGFR) [28,29]. In addition, integrin-targeting approaches utilizing antibodies [30], peptides [31], or sequence mutation [32] have generally been investigated in preclinical and clinical studies. In this study, for the first time we developed a talin-binding small molecule, KCH-1521, that modulates downstream signaling molecules (Figure 5) and suggested in vitro anti-angiogenic effects of KCH-1521 on HUVECs (Figure 3). Our results corroborate previous studies that talin interacts with integrin β3, and that the integrin αVβ3 subunit plays an essential role in angiogenesis, especially in endothelial cells [13,14,33]. Furthermore, Monkley et al. [17] showed that talin1 inactivation in mouse embryos resulted in severe hemorrhage, poor organization into vessels, and defects on in vivo angiogenesis. Based on these findings, we speculate that talin modulation through KCH-1521 could play a critical role in endothelial cells during in vivo neoangiogenesis.

Interestingly, we observed that *Dll4* was the most decreased gene among the various angiogenic genes, and simultaneously *Notch1* and *Notch4* gene expression also reduced by treating HUVECs with KCH-1521 (Figure 3A). Notch1 and Notch4 are expressed in endothelial cells and Dll4-Notch signaling is important for vascular development [34]. In addition, Dll4 is highly expressed in the tumor vasculature and is a negative regulator of tumor angiogenesis [35]. These finding suggest that talin-modulating KCH-1521 may have therapeutic potential to suppress tumor angiogenesis. Moreover, we also found significant decreases in the expression of cell adhesion molecules such as *VCAM-1* and *ICAM-1* (Figure 3A). These molecules are expressed in endothelial cells in response to inflammatory cytokines and facilitate leukocyte adhesion and infiltration in inflamed tissues [36]. Taken together, these results indicate that not only angiogenesis, but inflammatory responses could be affected by KCH-1521, indicating promising therapeutic potentials to treat various inflammatory disorders.

Nonetheless, there are a few limitations in the present study. First, we suggested in vitro cellular characterization of KCH-1521 by various methods, however, we did not perform in vivo evaluations of the pharmacokinetic or pharmacodynamics of KCH-1521. Second, we did not examine the direct effects of KCH-1521 on the integrin β3-talin interaction in the present study. Lastly, we investigated the effects of talin modulation by KCH-1521 using HUVECs, however, additional analyses using other subtypes of endothelial cells or several cell types that express talin1 and talin2 are required to verify our results.

To the best of our knowledge, this study is the first to demonstrate that talin modulation by novel KCH-1521 decreased the in vitro angiogenesis in HUVECs. Reduced expression of FA molecules and the downstream signaling molecules of talin induced increased apoptosis and decreased cell proliferation and in vitro angiogenesis. Further in vivo studies and optimization of KCH-1521 may lead to talin-targeted anti-angiogenic drugs for the treatment of vascular diseases and cancer.

## 4. Materials and Methods

### 4.1. Synthesis of N-((2-(1H-Indol-3-yl)ethyl)carbamoyl)-2-(benzo[d][1,3]dioxol-5-yloxy)acetamide (KCH-1521)

A mixture of 2-chloroacetamide (0.9 g, 10.69 mmol), sesamol (1.4 g, 10.69 mM), NaI (2.4 g, 16.03 mmol), and K_2_CO_3_ (4.43 g, 32.07 mmol) in *N*,*N*-dimethylformamide (DMF; 21 mL) was refluxed for 24 h. The resulting mixture was filtered with ethyl acetate (EtOAc; 30 mL). The organic mixture was washed with water (10 mL × 3) and dried over anhydrous MgSO_4_. Volatiles were removed under reduced pressure. The remaining residue was separated with SiO_2_ chromatography (EtOAc:*n*-hexane = 1:1) to give 2-(benzo[*d*][1,3]dioxol-5-yloxy)acetamide as a brown solid (0.87 g, 42%): proton nuclear magnetic resonance (^1^H-NMR; 500 MHz, DMSO (D8418, Sigma-Aldrich, St. Louis, MO, USA)-*d*_6_) δ 7.47 (1H, s), 7.37 (1H, s), 6.81 (1H, d, *J* = 10.0 Hz), 6.65 (1H, d, *J* = 5.0 Hz), 6.38 (1H, dd, *J* = 2.5 Hz, 8.5 Hz), 5.96 (2H, s), 4.32 (2H, s); carbon-13 NMR (^13^C-NMR; 500 MHz, DMSO-*d*_6_) δ 170.0, 153.1, 147.8, 141.5, 107.9, 106.0, 101.1, 98.1, 67.6; IR (ATR-IR) 3409, 3130, 1685, 1188 cm^−1^; low resolution mass spectroscopy (LRMS) [M + Na]^+^ 218.

A solution of the above acetamide (60 mg, 0.34 mmol) and oxalyl chloride (0.04 mL, 0.51 mmol) in dichloroethane (DCE; 1.7 mL) was stirred at 85 °C for 5 h. The resulting mixture was concentrated under reduced pressure. The remaining residue was dissolved in dichloromethane (DCM) and treated with tryptamine (55 mg, 0.34 mmol). After stirring for 3 h, distilled water was added. The mixture was extracted with DCM and dried over MgSO_4_. Volatiles were removed under reduced pressure. The remaining residue was separated with SiO_2_ chromatography (EtOAc:*n*-hexane = 1:2) to give the title compound, *N*-((2-(1*H*-indol-3-yl)ethyl)carbamoyl)-2-(benzo[*d*][1,3]dioxol-5-yloxy)acetamide as a yellow solid (65 mg, 50% in 2 steps): ^1^H-NMR (500 MHz, DMSO-*d*_6_) δ 10.81 (1H, s), 10.26 (1H, s), 7.56 (1H, d, *J* = 7.5 Hz), 7.33 (1H, d, *J* = 8.0 Hz), 7.15 (1H, s), 7.06 (1H, t, *J* = 7.5 Hz), 6.96 (1H, t, *J* = 7.0 Hz), 6.80 (1H, d, *J* = 8.5 Hz), 6.64 (1H, d, *J* = 2.5 Hz), 6.33 (1H, dd, *J* = 2.5 Hz, 11.0 Hz), 5.96 (2H, s), 4.61 (2H, s), 3.46 (2H, q, *J* = 7.4 Hz), 2.88 (2H, t, *J* = 7.0 Hz); ^13^C-NMR (500 MHz, DMSO-*d*_6_); 169.63, 152.73, 152.30, 147.59, 141.34, 135.98, 126.79, 122.46, 120.66, 118.00, 117.93, 111.06, 107.64, 105.47, 100.82, 97.74, 66.67, 24.88; IR (ATR-IR) 3362, 1682, 1488, 1185 cm^−1^; LRMS [M + Na]^+^ 404.

### 4.2. Surface Plasmon Resonance (SPR)

SPR response curves were measured using a series of concentrations of the tested compound. A ProteOn XPR36 system (Bio-Rad Laboratories, Hercules, CA, USA) equipped with a sensor chip was used for real-time binding studies. Phosphate-buffered saline (PBS; 17-517Q, Lonza, Walkersville, MD, USA) with 0.005% Tween 20 (P2287, Sigma-Aldrich) was used as the assay running buffer and for sample preparation. Bacterially expressed talin was immobilized onto channels of the HTE chip (Bio-Rad Laboratories), leaving one channel as a reference. A 210 μL sample of talin (2.5 µg/mL, His-tagged) was injected into the flow cells (30 μL/min) to allow saturation of the HTE chip by talin. To perform binding kinetics analysis, 100 μL aliquots of serially diluted compounds ranging from 50 to 800 μM with 4% DMSO in 1× tris-buffered saline (TBS; WH400028806, 3M, Maplewood, MN, USA) + 0.1% Tween 20 (TBST) were injected and allowed to flow through the channels of the HTE chip for 1 min at 100 μL/min. Relationships between the RU were obtained, and the concentrations of the tested compounds were plotted. To obtain dissociation curves, the analyte injection was stopped, and PBST (100 μL/min) was flowed over the chip for 200 s to dissociate the bound analytes. The ProteOn XPR36 control software ProteOn Manager v.3.1.0.6 (Bio-Rad Laboratories) was used to record changes in RUs, plot the binding curves, and analyze the curves. The RUs were normalized by subtracting the RUs of the empty channel. The kinetic and equilibrium constants were obtained based on a global fit, using the Langmuir 1:1 bimolecular kinetic model.

### 4.3. Cell Sources

HUVECs (PCS-100-010, ATCC) and NHDFs (CC-2509, Lonza) as a control for talin abundance were purchased and maintained in endothelial cell growth medium (EGM-2MV; CC-3202) or fibroblast growth medium (FBM-2; CC-3132, both from Lonza). Both culture media contained 100 U/mL penicillin/streptomycin (P/S; #15140, Gibco, Grand Island, NY, USA). All cells were incubated at 37 °C in a humidified incubator with 5% CO_2_ and used at passage 7 to 10. Culture media were changed every two days. In experiments using KCH-1521, HUVECs were incubated with either KCH-1521 at a concentration of 100 μM or the equivalent amount of DMSO (1% (*v*/*v*)) as a vehicle control in culture medium. The cell length in the long axis and cell numbers were measured by phase-contrast images using the ImageJ (ver. 1.49) program (National Institute of Health, Bethesda, MD, USA).

### 4.4. WST-1 Proliferation Assay

HUVECs were plated at a density of 8 × 10^3^ cells/well in 96-well plates to analyze cell proliferation with the tetrazolium salt WST-1 (05015944001, Roche Applied Science, Mannheim, Germany). Briefly, the cells were treated with eight different concentrations of KCH-1521 (0, 3.125, 6.25, 12.5, 25, 50, 100, and 200 μM) or the equivalent amount of DMSO in each concentration for 24 h, and incubated at a concentration of 10 μM of WST-1 reagent for 2 h until color development was sufficient for photometric detection. The reaction product was quantified by measuring the absorbance at 440 nm using a microplate reader (M2e, Molecular Devices, Sunnyvale, CA, USA), and the reference wavelength was 600 nm. Data were analyzed using quantification of absorbance analysis of the SoftMax Pro software (Molecular Devices).

### 4.5. Cell Adhesion Assay

For detection of live cells, nuclei of HUVECs were stained with NucBlue^®^ Live (live DAPI; R37605, Life Technologies, Eugene, OR, USA) for 20 mins according to the manufacturer’s instructions. The cells were seeded in fibronectin (F0895, Sigma-Aldrich)-coated six-well plates at a density of 5 × 10^4^ cells/well with either DMSO or KCH-1521-containing culture medium. After 10 mins of attachment, the unattached cells were washed twice with 1× PBS and fixed with 2% paraformaldehyde (PFA; P6148, Sigma-Aldrich). All images were taken immediately using an upright fluorescence microscope (DMI 3000B, Leica Microsystems, Wetzlar, Germany) and the live adherent cells were counted in five different fields per well.

### 4.6. Immunofluorescence Staining

HUVECs were fixed with 2% PFA after treatment for 24 h with DMSO or KCH-1521, and blocked with 5% normal goat serum (NGS; #16210, Gibco) in 1× PBS + 0.1% Tween 20 (PBST) for 1 h. The cells were incubated for 1 h at room temperature (RT) with the following primary antibodies: talin (T3287), vinculin (V9131, both from Sigma-Aldrich), and paxillin (610051, BD Biosciences, San Jose, CA, USA). After washing, the cells were incubated for 1 h at RT with Alexa Fluor 594-conjugated anti-mouse IgG (A11005) and Alexa Fluor 488-conjugated Phalloidin (A12379, both from Molecular Probes, Eugene, OR, USA). Nuclei were stained with 4′,6-diamidino-2-phenylindole (DAPI; D9542, Sigma-Aldrich). Negative controls for immunofluorescence staining were used to evaluate the specificity of primary antibodies and to exclude the possibility of non-specific staining of secondary antibodies by omitting the incubation of primary antibodies [37]. All immunofluorescent images were obtained using the TE-FM Epi-fluorescence system attached to an inverted microscope (BX61, Olympus, Tokyo, Japan).

### 4.7. Apoptosis Assay

After 24 h of treatment with DMSO or KCH-1521, the cells were fixed with 2% PFA and blocked with 5% NGS. Briefly, the cells were incubated with cleaved caspase-3 (#9661, Cell Signaling Technology, Danvers, MA, USA) and Alexa Fluor 594-conjugated anti-rabbit IgG (A11012, Molecular Probes). Nuclei were stained with DAPI, and immunofluorescence images were obtained using the TE-FM Epi-fluorescence system attached to an inverted microscope.

Annexin V and PI staining was performed using the FITC Annexin V apoptosis detection kit I (556547) according to the manufacturer’s instructions, and then 3 × 10^4^ cells were analyzed using a flow cytometer (FACS Calibur) and Cell Quest Pro software (all from BD). Annexin V-negative and PI-negative cells were identified as viable, Annexin V-positive and PI-negative cells as early apoptotic, Annexin V-positive and PI-positive cells as late apoptotic, and Annexin V-negative and PI-positive cells as necrotic cells.

### 4.8. In Vitro Tube Forming Assay

HUVECs were plated on Matrigel (356230, BD)-filled 12-well plates at a density of 3 × 10^3^ cells/well and incubated in culture medium. After 12 h, the culture medium was changed to EGM-2MV containing either DMSO or KCH-1521. After 6, 12, 24, and 72 h of treatment, in vitro tube formation was observed. All phase-contrast images were acquired using an upright fluorescence microscope (DMI 3000B, Leica Microsystems).

### 4.9. Real-Time Polymerase Chain Reaction (PCR)

After 24 h of treatment with DMSO or KCH-1521, total RNA was extracted from cells using TRI Reagent (TR118, MRC, Cincinnati, OH, USA), and the concentration of total RNA was measured using a Nanodrop 1000 spectrophotometer (Thermo scientific, Waltham, MA, USA). Synthesized cDNA was then analyzed by real-time PCR using iQ^TM^ SYBR Green Supermix and the My iQ™ 2 real-time PCR detection system (both from Bio-Rad Laboratories) using anti-human primers. The primer sequences used for real-time PCR are listed in Supplementary Table S1. To avoid genomic DNA amplification, intron-spanning primers were designed using Probe Finder software (https://www.roche-applied-science.com). Relative gene expression levels were normalized to glyceraldehyde 3-phosphate dehydrogenase (*GAPDH*) as a reference gene and presented as relative expression to the control using the ddCt method.

To perform semi-quantitative PCR, cDNA was synthesized from the extracted RNA. Initially, cDNA was analyzed using the real-time PCR as mentioned above. After amplification, the PCR reaction products were loaded onto 10% agarose gels for electrophoresis. The intensity of each band was quantified using a gel imaging system (GelDoc XR, Bio-Rad Laboratories).

### 4.10. Western Blot

HUVECs were treated for 24 h with DMSO or KCH-1521 after 24 h of incubation. Cell lysates were prepared using ice-cold 1× cell lysis buffer (#9803S, Cell Signaling Technology) with 1 mM phenylmethylsulfonyl fluoride (PMSF) for 1 h at 4 °C. Protein concentrations of samples were determined via Bradford protein assay (#500-0006, Bio-Rad Laboratories). Equal amounts of protein were separated by 10% sodium dodecyl sulfate (SDS)-polyacrylamide gel electrophoresis (SDS-PAGE) and transferred to a polyvinylidene difluoride (PVDF; 10600023, GE Healthcare, Chicago, IL, USA) membrane. The membranes were blocked with 5% skim milk in TBST for 1 h at RT and incubated overnight at 4 °C with the following primary antibodies: talin (#4021, Cell Signaling Technology), vinculin, and paxillin. After washing with TBST, the membranes were stained with the appropriate horseradish peroxidase (HRP)-conjugated secondary antibodies: anti-rabbit (sc-2030) or anti-mouse (sc-2005, both from Santa Cruz Biotechnology, Santa Cruz, CA, USA). The bands were visualized using ECL plus (32132, Thermo Scientific) reagent and exposed to radiography films (EA8EC, AGFA). The protein levels were normalized to GAPDH (G8795, Sigma-Aldrich), and the intensity of bands was analyzed using Quantity One software (Bio-Rad Laboratories).

HUVECs were incubated on fibronectin-coated dishes for 6 h and treated with KCH-1521 for 4 h. Briefly, equal amounts of protein were subjected to SDS-PAGE and transferred as mentioned above. The membranes were blocked and incubated overnight at 4 °C with the following primary antibodies: AKT1 (sc-1618, Santa Cruz), FAK (#13009), phospho-FAK (Tyr^397^) (#8556), phospho-AKT (Ser^473^) (#9271), p44/42 MAPK (ERK1/2) (#9102), and phospho-p44/42 MAPK (ERK1/2; #9106, all from Cell Signaling Technology). After washing, the membranes were stained with the appropriate HRP-conjugated secondary antibodies: anti-goat (HAF109, R&D Systems, Minneapolis, MN, USA), anti-rabbit, or anti-mouse. The protein levels were normalized to GAPDH, and the band intensity was analyzed.

### 4.11. Statistical Analysis

All values are presented as mean ± standard deviation (SD). Comparison among values for the analysis of differences between groups was performed by Student’s *t*-test. Analysis of variance (ANOVA) followed by Student-Newman Keuls test was used to compare the values for all groups. All experiments were repeated at least three times, and * *p* < 0.05 was considered to be statistically significant. 

## Supplementary Materials

Supplementary materials can be found at www.mdpi.com/1422-0067/18/1/221/s1.
