# Supplementary material for: Talin Modulation by a Synthetic N-Acylurea Derivative Reduces Angiogenesis in Human Endothelial Cells
Source: Int J Mol Sci. 2017 Jan 22;18(1):221. doi: 10.3390/ijms18010221 (PMC5297850; doi:10.3390/ijms18010221)
Supplement: Supplementary file 1 [file ijms-18-00221-s001.pdf]

Supplementary Materials: Talin Modulation by a Synthetic *N*-acylurea Derivative Reduces Angiogenesis in Human Endothelial Cells

I-Rang Lim, Hyung Joon Joo, Minseon Jeong, Jong-Ho Kim, Seung-Cheol Choi, Chungho Kim, Jong-Wha Jung and Soon Jun Hong

Table S1. Primer information for real-time PCR.

Gene	Primer Sequence
CD31	5′-GAGTCCTGCTGACCCTTCTG<br>3′-TCAGGTTCTTCCCATTTTGC
Vascular endothelial-Cadherin (*VE-Cadherin*)	5′-GGCATCTTCGGGTTGATCCT<br>3′-CCGACAGTTGTAGGCCCTGTT
von Willebrand factor (*vWF*)	5′-TAAGTCTGAAGTAGAGGTGG<br>3′-AGAGCAGCAGGAGCACTGGT
Tie2	5′-TTGAAGTGGAGAGAAGGTCTG<br>3′-GTTGACTCTAGCTCGGACCAC
Vascular cell adhesion molecule-1 (*VCAM-1*)	5′-TGCACAGTGACTTGTGGACAT<br>3′-CCACTCATCTCGATTTCTGGA
Intercellular adhesion molecule-1 (*ICAM-1*)	5′-CCTTCCTCACCGTGTACTGG<br>3′-AGCGTAGGGTAAGGTTCTTGC
C-X-C chemokine receptor type 4 (*CXCR4*)	5′-CCTGCCTGGTATTGTCATCC<br>3′-AGGATGACTGTGGTCTTGAGG
CD34	5′-TGGAGCAAAATAAGACCTCCAG<br>3′-AAGGAGCAGGGAGCATACC
Fibroblast growth factor-2 (*FGF-2*)	5′-AGCGGCTGTACTGCAAAAAC<br>3′-GCTTGAAGTTGTAGCTTGATGTG
Apelin	5′-ATAAGGGACCCATGCCTTTC<br>3′-CCTCCAGAGAAGCAGACCAA
Urokinase receptor (*uPAR*)	5′-GTAGCCACCGGCACTCAC<br>3′-TGGTCTCAGGGCAGTAGTACTTT
Endothelial nitric oxide synthase (*eNOS*)	5′-GACTGAAGGCTGGCATCTG<br>3′- CCATGTTACTGTGCGTCCA
Neuropilin 2 (*NRP2*)	5′- CTGAACAAGCTCCACGCTCCACTG<br>3′- CCTGAGAGCATCCCCAGCATGTTG
Roundabout guidance receptor 4 (*ROBO4*)	5′-GCATCATCCGTGGCTACC<br>3′-ACAGTCCAGTTGGCTGGTG
Delta-like ligand 4 (*Dll4*)	5′-GTTTCCCCACAGTGACAAGAGCTTAG<br>3′-CCTTATACCTCCGTGGCAATGACAC
Notch1	5′-CAATGACCCCTGGAAGAACTGC<br>3′-CTTCCGCACGCTGGCAGTCAAAG
Notch4	5′-AGAAAGACAAGGCCAACTGC<br>3′-CACGTCACACACACATGAGG
Vascular endothelial growth factor receptor 1 (*VEGFR1*)	5′-CCAGCAGCGAAAGCTTTGCG<br>3′-CTCCTTGTAGAAACCGTCAG
Vascular endothelial growth factor receptor 2 (*VEGFR2*)	5′-ATGACATTTTGATCATGGAGC<br>3′-CCCAGATGCCGTGCATGAG
Vascular endothelial growth factor receptor 3 (*VEGFR3*)	5′-TGCAAGAGGAAGAGGAGGTCT<br>3′-CAGGCTTGGCGGGCTGTGC
Paxillin	5′-CAGCAGACACGCATCTCG<br>3′-GAGCTGCTCCCTGTCTTCC
Talin1	5′-ACCAGTGACTATGGCCGTCT<br>3′-CGGTGTTTGATATGGGAACC
Talin2	5′-GGCCCAGGATGATCTCAGT<br>3′-TCTGAACCCATACCCTAGATGC
Glyceraldehyde 3-phosphate dehydrogenase (*GAPDH*)	5′-GAGTCCACTGGCGTCTTCAC<br>3′-TTCACACCCATGACGAACAT

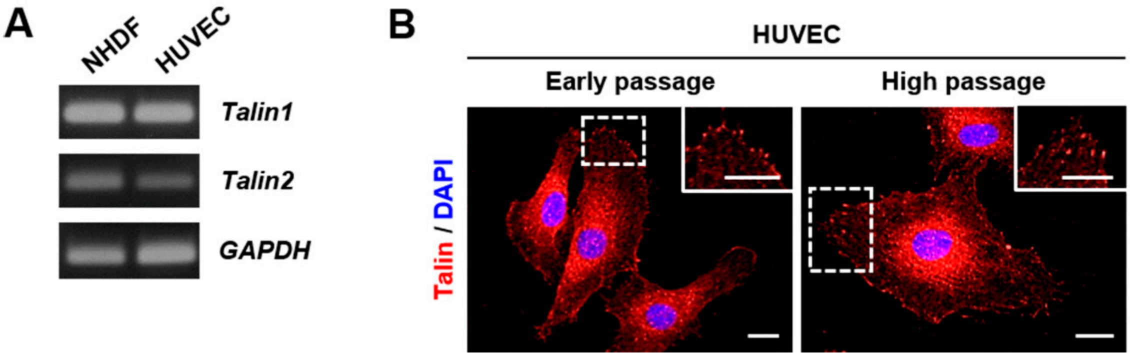

Figure S1. Positive expression of talin in human umbilical vein endothelial cells (HUVECs). (**A**) Semi-quantitative PCR analysis of *talin1* and *talin2* expressions in normal human dermal fibroblasts (NHDFs) and HUVECs; (**B**) representative immunofluorescence images of talin (red) expression in the early passage (passage 7, left) and in the high passage of HUVECs (passage 15, right). The magnified images showing talin expression in focal adhesion (FA). Nuclei were stained with DAPI (blue). Scale bars = 20 μm.
